# Supplementary material for: Combination Regimens of Favipiravir Plus Interferon Alpha Inhibit Chikungunya Virus Replication in Clinically Relevant Human Cell Lines
Source: Microorganisms. 2021 Feb 2;9(2):307. doi: 10.3390/microorganisms9020307 (PMC7912990; doi:10.3390/microorganisms9020307)
Supplement: Supplementary file 1 [file microorganisms-09-00307-s001.pdf]

microorganisms

**Supplemental material**

*FAV Triphosphate Assay*

HT-1080, SK-N-MC, and HFF-1 cells were treated with various concentrations of FAV ranging from 0 to 157.10 μg/ml for one day. Cells were harvested and separated from drug containing medium by centrifugation and washed twice with PBS to ensure removal of extracellular FAV. FAV and FAV-RTP were quantified via LC-MS/MS. Our system consisted of a 1260 HPLC (Agilent) and a 6460 triple quadrupole mass spectrometer (Agilent). Protein precipitation was achieved by Trichloroacetic acid (TCA). The supernatant was obtained by centrifugation and $NH_4OH$ was used to adjust the pH to a weak base. The FAV was analyzed using a Kinetex 2.6 μm Polar C18 LC column, 50 × 2.1mm. Mobile phases consisted of 0.1% Formic acid in water (A) and methanol (B) at a flow rate of 0.4 ml/min in gradient mode. The FAV-RTP was analyzed using a hypercarb 5 μm LC column, 50 × 4.6mm. Mobile phases consisted of 0.1% $NH_4OH$ in water (A) and ACN/IPA (1:3) (B) at a flow rate of 0.4 ml/min in gradient mode. The mass transition (MRM) of FAV m/z 156 → 113 in positive ion mode, and FAV-RTP m/z 528 → 159 in negative ion mode.

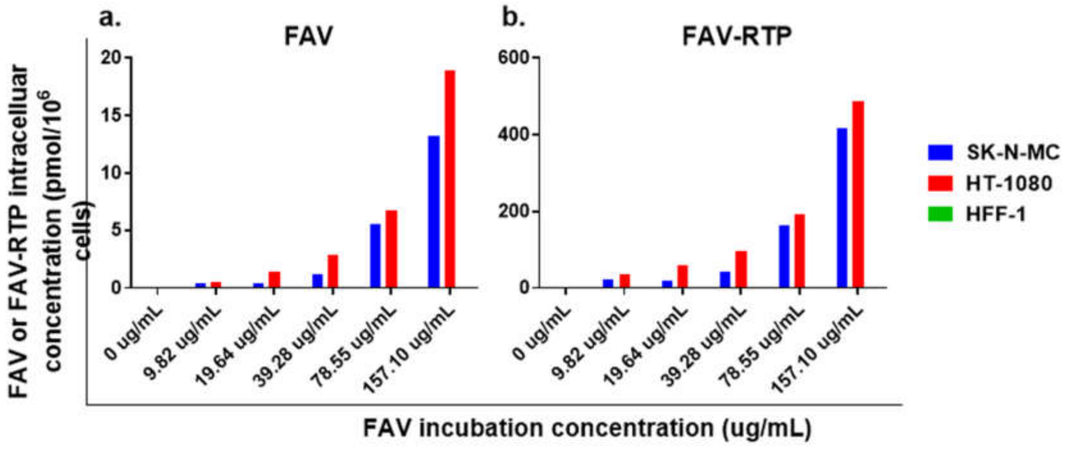


**Supplemental Figure 1:** Intracellular concentrations of FAV (a) and FAV-RTP (b) in HT-1080, SK-N-MC, and HFF-1 cells. Cells were incubated with increasing concentrations of FAV ranging from 0 to 157.1 μg/ml for one day then extracellular FAV was removed and intracellular FAV and FAV-RTP concentrations were quantified via LC-MS/MS.
